# The dynamic influence of human resources on evidence-based intervention sustainability and population outcomes: an agent-based modeling approach

**DOI:** 10.1186/s13012-018-0767-0

**Published:** 2018-06-05

**Authors:** Virginia R. McKay, Lee D. Hoffer, Todd B. Combs, M. Margaret Dolcini

**Affiliations:** 1 Center for Public Health Systems Research in the Warren G. Brown School of Social Work, Washington University in St. Louis, Campus Box1196, One Brookings Drive, St. Louis, MO 63130 USA; 2 Department of Anthropology, Case Western Reserve University, Mather Memorial Room 238 11220, Bellflower Road, Cleveland, OH 44106-7125 USA; 3 School of Social and Behavioral Health Sciences, College of Public Health and Human Sciences, Oregon State University, Hallie E. Ford Center for Health Children and Families, 2631 SW Campus Way, Corvallis, OR 97331 USA

**Keywords:** Sustainability, Agent-based modeling, Evidence-based intervention, Human resources, Dissemination and implementation science, Organizational capacity, Systems science

## Abstract

**Background:**

Sustaining evidence-based interventions (EBIs) is an ongoing challenge for dissemination and implementation science in public health and social services. Characterizing the relationship among human resource capacity within an agency and subsequent population outcomes is an important step to improving our understanding of how EBIs are sustained. Although human resource capacity and population outcomes are theoretically related, examining them over time within real-world experiments is difficult. Simulation approaches, especially agent-based models, offer advantages that complement existing methods.

**Methods:**

We used an agent-based model to examine the relationships among human resources, EBI delivery, and population outcomes by simulating provision of an EBI through a hypothetical agency and its staff. We used data from existing studies examining a widely implemented HIV prevention intervention to inform simulation design, calibration, and validity. Once we developed a baseline model, we used the model as a simulated laboratory by systematically varying three human resource variables: the number of staff positions, the staff turnover rate, and timing in training. We tracked the subsequent influence on EBI delivery and the level of population risk over time to describe the overall and dynamic relationships among these variables.

**Results:**

Higher overall levels of human resource capacity at an agency (more positions) led to more extensive EBI delivery over time and lowered population risk earlier in time. In simulations representing the typical human resource investments, substantial influences on population risk were visible after approximately 2 years and peaked around 4 years.

**Conclusions:**

Human resources, especially staff positions, have an important impact on EBI sustainability and ultimately population health. A minimum level of human resources based on the context (e.g., size of the initial population and characteristics of the EBI) is likely needed for an EBI to have a meaningful impact on population outcomes. Furthermore, this model demonstrates how ABMs may be leveraged to inform research design and assess the impact of EBI sustainability in practice.

**Electronic supplementary material:**

The online version of this article (10.1186/s13012-018-0767-0) contains supplementary material, which is available to authorized users.

## Background

Evidence-based interventions (EBIs) are intended to help ensure beneficial outcomes for the individuals and communities that receive them and have been widely implemented in public health practice. Dissemination and implementation in research is dedicated to understanding the factors that influence the dissemination and implementation of EBIs [1]. Although many EBIs are adopted and initially implemented, adequately delivering and sustaining an EBI is an ongoing challenge in practice [2–4]. Organizational capacity, especially adequate staffing, is commonly reported as a contributing factor to whether or not an EBI is sustained. Without adequate staffing, EBIs may not be appropriately provided to meet community need (e.g., insufficiently available or underused) or may be prematurely abandoned altogether. Furthermore, inadequate EBI delivery may not have demonstrable effects on the intended population outcomes. While there is need to identify the appropriate level of staffing to bolster the successful delivery and sustainability of EBIs in practice, a particular challenge of empirically examining these issues is the longitudinal and dynamic nature between adequate staffing and EBI sustainment [5, 6]. We use agent-based modeling, a computational systems science approach, in conjunction with existing EBI implementation frameworks and empirical data, to examine the relationships between staffing, sustainability, and population health.

### Organizational capacity, EBI sustainability, and population outcomes

**Fig. 1:**
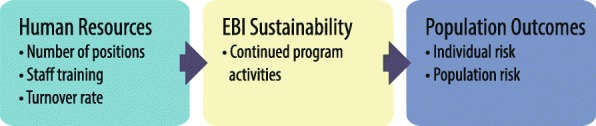
ABM variables using organizational capacity model developed by Meyer et al. [7] and integrated with EBI sustainability model by Scheirer and Dearing [11]

We frame the relationship between human resources, EBI sustainability, and population outcomes with the general premise that the level of human resources influences the extent to which organizations deliver and sustain an intervention, which, in turn, influences how the intervention impacts the intended population. These relationships are conveyed in Fig. 1. Human resources fall within the conceptual domain of organizational capacity, describing the resources available to public health organizations to deliver essential services and improve population health [6, 7]. Public health organizations require adequate capacity to sustain services at appropriate levels and meet community needs over time. The organizational capacity model developed by Meyer, Davis, and Mays provides a framework for organizational capacity and services [7]. This model is grounded in organizational theory for health organizations [8, 9] and empirical evidence [10], and it suggests that the level of capacity within an organization influences the services it provides by the organization. The organizational capacity model incorporates multiple components that comprise capacity (e.g., financial, human, physical, and informational resources) and identifies specific variables to operationalize and measure each component. Human resources are identified as a major, and perhaps the most vital, component of organizational capacity and are operationalized as number of full-time employees, staff knowledge and skills, education experience, training, staffing configuration, retention/turnover, and compensation.

To complement our use of the organizational capacity model and its exposition of human resources, Scheirer and Dearing’s conceptual framework of EBI sustainability identifies variables for measuring sustainability. This model defines sustainability as the final stage of program implementation when programs are maintained and become integrated into the regular functioning of an organization [11]. Both models hypothesize a relationship between available human resources, EBI sustainability, and downstream community benefits. If the EBI is no longer benefiting the community as a whole, then it may need to be adapted, discontinued, and/or replaced to better align with community need.

### Challenges to examining EBI sustainability

Identifying essential resource components, for example minimum numbers of staff dedicated to an intervention, should ideally be part of assessing EBI delivery, sustainment, and intended outcomes. However, examining the relationships between human resources and EBI implementation over extended periods of time is difficult using common research designs. EBI sustainability occurs over relatively longer time periods than earlier implementation phases (e.g., adoption and early implementation). During the sustainability phase, EBI activities ideally persist despite common changes in the agency, like staff turnover [12]. Using longitudinal designs—following organizations over extended periods of time and collecting large volumes of quantitative data—is costly and impractical, making capturing relationships between human resources and EBI sustainability difficult [13]. Additionally, researchers often only assess the influence of EBI delivery on direct recipients of services but cannot capture the impact of EBI delivery for entire communities. Qualitative designs are used to examine organizational capacity and EBI implementation because they offer the necessary detail that is required to explain the relationships that may influence EBI sustainability [14, 15], but the ability to generalize results from these studies across different contexts is limited.

### Agent-based modeling to examine sustainability

Agent-based modeling (ABM) is one approach that offers several advantages in examining EBI sustainability. ABMs model the behavior of heterogeneous individuals (i.e., agents) and the interaction between them in an environment. The aggregated individual behaviors of agents illuminate the dynamics of the larger social structures they both comprise and create [16]. Simulations can be constructed using existing empirical data and then manipulated to produce alternative outcomes. These models can also inform hypothesis generation, guide future data collection, and inform theory development. In this way, ABM complements both qualitative and quantitative research approaches by incorporating details of context while providing a virtual lab to generate plausible explanations and descriptions of relationships among variables over a lengthy period of time [17]. A small number of ABMs have been developed to assess the impact of public health policy and services in communities [18–21], organizational behavior [22], and EBI implementation for disease prevention [23]. These ABMs show promise in their ability to demonstrate the impact of EBIs on population health but have been underutilized in dissemination and implementation research [24].

### The current study

The strengths of ABM provide an opportunity to examine the sustainability of EBIs in public health organizations over time. The approach may also offer insights into factors and dynamics influencing EBI sustainability that are otherwise difficult to obtain. Guided by the theoretical premise that optimal human resources lead to more sustainable EBIs and greater improvements in population health, we use ABM to explore the relationship among human resources, EBI sustainability, and population outcomes to inform theoretical models of organizational capacity and demonstrate the use of ABM to address pressing EBI sustainability research questions.

## Methods

Our ABM models the delivery of an EBI by staff to a population in a hypothetical agency over a period of years. Initially, the model creates an agency with staff hired to provide the intervention and the population. As the simulation moves forward in time, staff are trained and deliver the EBI to a population at risk for human immunodeficiency virus (HIV). As staff leave the agency, the agency hires and trains new staff to fill the number of available positions. We assessed the influence of human resources by systematically adjusting three key human resource variables: the number of staff positions at the agency, the rate of turnover, and the length of time required for training newly hired staff for EBI delivery.

### Data sources

We used the organizational capacity model developed by Meyer, Davis, and Mays; the EBI sustainability model by Scheirer and Dearing [7, 11] (see Fig. 1); and data from existing studies focusing on the EBI, RESPECT, to inform our ABM (see Table 1). RESPECT is a brief, evidence-based counseling and testing intervention that aims to reduce sexually transmitted infections and HIV by reducing high-risk sexual behaviors that make infection transmission more likely [25, 26]. As originally designed, RESPECT is delivered in a two-session format where clients identify risk behaviors and develop a plan for reducing identified behaviors in the first counseling session. In the second session, clients discuss their success or failure in achieving their risk-reduction plan and identify additional behaviors to help support and improve behavioral changes. RESPECT was supported by the Centers for Disease Control and Prevention (CDC) and was one of several EBIs widely implemented for reduction of sexually transmitted infections in local settings [27].

**Table 1:** Key simulation variables and parameters

	Variable	Description	Data source(s)^a^	Value
1.1	Loss to follow up (%)	The proportion of clients that receive the first session of RESPECT but will not return for the second session	RCT	15
1.2	Risk reduction achieved (%)	The proportion of clients that will achieve their risk-reduction step	RESPECT case	72
1.3	Size of risk reduction (M; SD)	The size of the risk-reduction step achieved by the clients selected from normal distribution	RCT; RESPECT case	1; 0.5
1.4	Clients in a week (N)	The number of clients seen by one provider in a week	Project RESPECT	15
1.5	Risk decay (%)	The proportion of clients that initially achieved their risk-reduction step but experience an increase in risk	RCT	8
1.6	Size of risk decay (M; SD)	The size of the risk-reduction step achieved by the clients selected from a normal distribution	RCT; RESPECT case	1; 0.5
1.7	Repeat eligibility criteria (Months; Risk)	The criteria determining whether an individual in the population can participate in the intervention again	Project RESPECT	3; < 4

^a^Project RESPECT  = data from translation of project RESPECT (see references [28–30]). RCT = data from the original RESPET randomized controlled trial (see references [24, 25]). RESPECT case = data from the RESPECT de-adoption study (see reference [31])

We used published statistics from the original RESPECT randomized-control trial [25, 26] and CDC publications characterizing standard program structure and protocols [28] to inform overall model design. We also used data from two RESPECT implementation studies to inform the characterization of human resources and acquire data on RESPECT clients, staff, and agencies [29–32]. Specific variables, descriptions, values, and empirical data sources for each variable used to inform simulation runs are provided in Table 1.

### Model design and development

We used Netlogo 5.2 to develop, calibrate, validate, and execute the ABM [33]. Each simulation run begins by generating a number of locations where the EBI is delivered, a provider population, and a client population. A screen shot of the simulation view and code are provided in Additional file 1. The agency has a maximum number (from two to ten) of provider positions available. At the beginning of a simulation run, the agency hires the maximum number of staff to fill all available positions, which we refer to as staff positions. All newly hired staff begin as untrained.

The simulation also generates a population for recruitment to receive the EBI. There are limited data to support the number of individuals at risk for a sexually transmitted infection in any community. However, crude estimates of populations at increased risk for HIV, for example, give a very basic sense of the number of individuals at risk. For example, men who have sex with men, who make up 63% of all new HIV cases, make up approximately 2 % of the US population [34]. Presuming 2 % of the population is at increased risk for HIV, a community of approximately 500,000 would have a risk population of 10,000, and the target population size in our model was held constant at 10,000 individuals.

The population of potential clients represents the number of individuals in the population at increased risk for a sexually transmitted infection or HIV. Given the ongoing debate around the relationship between risk behaviors and actual risk of transmission [35], we represent risk of transmission (i.e., the likelihood that an individual will get HIV based on their behavior) as an abstract value rather than imply risk of transmission using specific risk behaviors as a proxy (i.e., sex without condoms or intravenous drug use). We selected a Poisson distribution of risk based on existing literature suggesting larger proportions of individuals in the USA are at relatively low risk for HIV transmission, while smaller proportions are at relatively high risk [36, 37]. We held the mean of the distribution constant at 2.5 at the start of a simulation for all simulation runs. We capped individual risk values at zero and seven for two reasons: (1) Individuals cannot exhibit less than zero risk (i.e., they have no risk) and (2) this places individuals on a natural risk scale where individuals on the lower end of the scale (i.e., less than three) can be interpreted as a low-risk group and individuals on the higher end of the scale (i.e., greater than five) can be interpreted as a high-risk group.

### Simulation runs

Over time, the agency has two basic sets of activities. The first is a set of activities representing regular, daily EBI delivery to clients by providers, and the second is a set of weekly activities assessing and updating available providers (i.e., the human resource capacity) at the agency.

#### Regular EBI delivery

Based on the availability of trained staff at the agency, clients are recruited from the target population for the EBI. Clients who have not yet received both sessions the EBI are randomly selected for recruitment from the target population and meet with an available provider. Following data from the literature discussed above (see Table 1), clients participate in the first session if they are new, and a proportion (15%) are lost to follow up and will not return for the second session. A proportion of the clients who return for a second session (72%) will achieve a risk-reduction step, and the individual risk value for each client is reduced. The size of the risk-reduction value is randomly selected from a normal distribution with a relatively modest mean and standard deviation. After receiving either session, all clients return to the general target population. The simulation loops through this set of procedures 15 times for each trained and available provider to represent a week’s worth of EBI delivery to clients, based on a reported average number of clients seen per week from RESPECT (see Table 1).

At the end of the week, clients that have received both EBI sessions and achieved their risk-reduction step may be randomly selected to experience behavior decay, and revert back to a higher risk level (see Table 1). Similar to risk reduction, the size of the risk increase is selected from a normal distribution with a relatively modest mean and standard deviation. Clients become eligible to participate in the EBI again after a 3-month period and if they are at exceptionally high risk for infection (i.e., a risk value over 4).

#### Weekly agency capacity

The simulation runs through a series of procedures to update the agency’s available providers. Again, following data from the literature discussed above (see Table 2), all newly hired providers receive training over a number of weeks (two to six). Once a provider is trained, the individual is available to provide the EBI to recruited clients during the week. Over time, providers have a probability (0.05 to 0.15) of leaving (i.e., turning over), and when this happens, a position at the agency then becomes available and an untrained provider is hired to fill the position. Each time step in the simulation represents 1 week of EBI delivery. The simulation ends after the equivalent of 10 years of agency operations (50 ticks = 1 year).

**Table 2:** Experimental conditions

Variable	Description	Range	Increment
Staff positions (N)	The maximum number of possible EBI staff positions at the agency	2–10	2
Turnover rate (%)	The proportion of providers that will turnover in a year	5–15	5
Timing in training (N)	The number of weeks that a provider will be present at the agency before being trained in the EBI	2–6	2

### Validation

We developed our model using best practices [38, 39]. All code was reviewed by an independent coder. We validated a baseline simulation of typical delivery of the EBI by using existing data. Baseline input parameters were the following: five provider positions, a turnover rate from 5 to 10% per year, and training 4 weeks after hired. Model outputs for our baseline model were calibrated to meet the following specific outcomes for clients after 1 year: a 15% loss to follow up, 89% achievement of risk-reduction step, and risk-reduction decay of 8% per year (see Table 1).

### Analysis

To identify the essential human resources to maximize impact on population risk, we conducted a set of experiments systematically varying the level of human resources. The simulation was run ten times for each combination of values of the experimental parameters in Table 2. For example, the simulation was run ten times with two available staff positions, a 5% yearly turnover rate, and a 2-week delay from the time a new individual was hired to the time that they were provided training. Analysis of model output data was performed using R 3.3.2 [40].

## Results

**Fig. 2:**
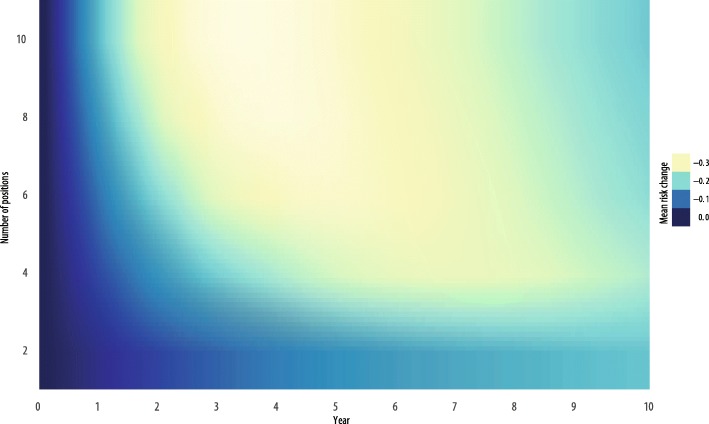
Contour map of change in population risk by staff positions over time

**Fig. 3:**
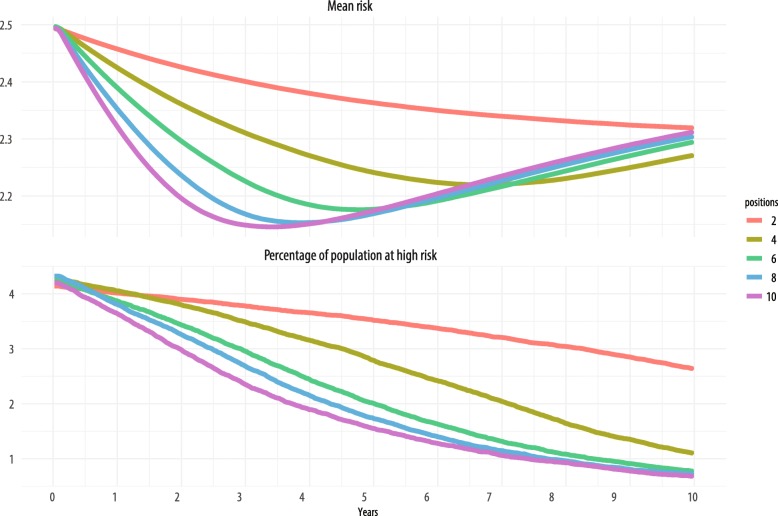
Trend graphs of the change in mean population risk and the proportion of the population considered high risk (risk > 5) over time

**Fig. 4:**
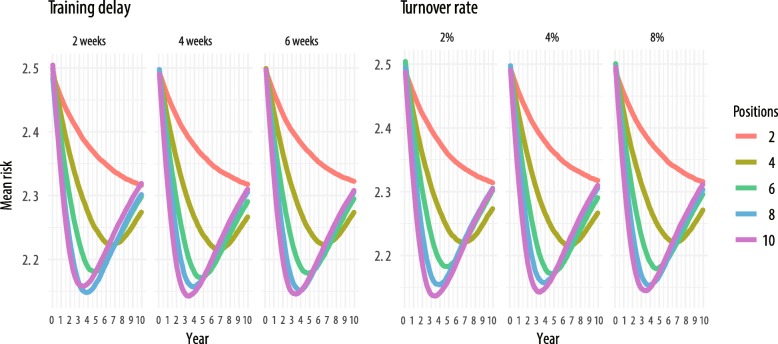
Training delays and turnover rates with mean population risk over time

Outcomes from simulation runs are presented in Figs. 2, 3, and 4. We observed the expected general relationship among human resources, EBI implementation, and population risk. Higher levels of human resources (e.g., more staff positions) resulted in more EBI delivery and greater decreases in population risk in a shorter amount of time. Conversely, lower overall levels of human resources resulted in less EBI delivery and limited influence on population risk, such that in some configurations, there was no apparent influence on population risk. Within these conditions, EBI implementation had little influence on population risk because the rate of delivery and achievement of risk reduction were either less than or equivalent to the combined rate of loss to follow up and behavior decay.

### Number of staff positions

Figure 2 presents a contour plot of simulation runs over the full 10 years. The primary human resource variable represented in the figure is the number of available staff positions dedicated to the EBI. The other two human resource variables, staff turnover and training, are held constant at mean values, 10% and 4 weeks, respectively. The *y*-axis is the mean risk of the population. The *x*-axis is time in years (0–10 years). Each line in the plot is the number of staff positions (2–10 positions) and is differentiated by color. Less saturated colors approaching white represent greater change in risk, and more saturated colors approaching black represent less change in risk. The plot demonstrates that simulation runs with two positions show a limited amount of influence on population risk at any time point. Among simulation runs with between four and eight positions, changes in population risk become visible around 2 years, with most simulation runs reaching peak change in population risk between 3 and 5 years. Interestingly, there is limited additional benefit in terms of population-level risk reduction among simulation runs with eight to ten positions relative to simulation runs with six positions.

The top panel in Fig. 3 shows the mean population risk over time for two, four, six, eight, and ten staff positions. Here again, the other two human resource variables are held constant at values identical to those in Fig. 2. Like in Fig. 2, we see that most of the largest decreases in risk come between 3 and 5 years into EBI implementation. However, for simulation runs with four staff positions, the largest decrease in risk is seen around 7 years, and for those runs with two providers, the minimum risk is observed at 10 years. More staff positions result in quicker rates of decrease in risk initially, but eventually risk levels out, and at 10 years, the mean risk for any number of staff positions centers around 2.3.

We also examined the influence of staff positions on EBI delivery over time on those at greatest risk and most in need of intervention. The bottom panel of Fig. 3 shows the percentage of population at high risk (risk > 5) on the *y*-axis for the same numbers of staff positions as in the top panel (with staff turnover and training still constant). Similar to outcomes for the overall population risk, simulations with only two staff dedicated to the EBI showed the least influence on the population proportion at increased risk. In contrast to the outcomes for the mean risk in the overall population, under EBI implementation with more than two staff positions, observable benefits in decreasing the proportion of the population at high risk continued throughout the 10-year period. Of particular note, we did not observe a floor effect—rather, individuals at increased risk relative to the rest of the population continued to benefit from the EBI throughout the entire 10-year simulated period.

### Training and turnover

Figure 4 shows the mean population risk (*y*-axis) over the 10-year period (*x*-axis), and each panel represents results at a different value of training time or turnover rate. The first set of three panels shows the risk by number of staff positions for the three unique values of staff training times (2, 4, and 6 weeks) holding turnover constant at its middle value of 10%. We see little or no discernable difference between the simulation run results when training time varies, e.g., the difference in mean risk at 10 years with ten staff members for a 2-week and a 6-week training time is approximately 0.02. We see similar results as the annual turnover rate varies from 5 to 15% in the three panels on the right (holding training time constant at 4 weeks). Investigation of potential multiplicative effects, e.g., in simulation runs with the maximum training time *and* maximum turnover rate, similarly showed no substantial impact when controlling for number of staff positions.

## Discussion

We explored the influence of human resources on EBI delivery over time and risk at the population level. Using an ABM based on available empirical evidence, we observed many of the theoretically supported relationships among human resources, EBI sustainability, and population impact [7, 11]. We also were able to explore and describe many of the dynamic interactions among these variables that would otherwise be difficult to examine. The outcomes from our ABM have several practical implications for assessing sustainability of EBIs and their impact on population health.

### Human resource, EBIs, and population dynamics

As the organizational capacity model suggests [7], overall levels of human resources influenced the extent of EBI sustainability and the impact of the EBI on population risk. As expected, a minimum human resource investment was necessary for the EBI to have important population benefits. Sufficient numbers of practitioners were needed to adequately deliver the EBI to an adequate proportion of the target population, especially given the likelihood that not all individuals will completely participate in the EBI (i.e., some will be lost to follow up) or maintain the benefits of the EBI (i.e., some will reverse achieved behavior changes). This reflects the reality of contextual factors that influence EBI success and sustainability. It also suggests that organizations attempting to implement EBIs without adequate capacity may see individuals reap benefits in reduced risk, but not see benefits in the overall population (i.e., reduced mean risk). Our ABM suggests that achievement of population risk reduction is contingent on the context in which an EBI is implemented, including the size of the target population, the level of organizational capacity supported over time, and the characteristics of EBI delivery.

This ABM also supports empirical evidence that smaller agencies, or agencies that are only able to dedicate a few staff to a particular service, have more difficulty sustaining EBIs and achieving population benefits [29, 41]. While timing in training and turnover had little impact on population outcomes relative to the absolute number of staff, these results should be interpreted with caution. For example, turnover, especially at higher levels, can impact service quality and drive up costs in staff replacement and training [42]. Moreover, we did not model financial resources, another major component of organizational capacity and a major predictor of EBI sustainability [7, 11], which may be a valuable extension of this model for others seeking to examine the dynamic relationships among organizational capacity variables.

### Assessing EBI sustainability and population benefit

The ABM also has implications for empirical examinations of human resources, EBI sustainability, and population outcomes. We used the Scheirer and Dearing framework of EBI sustainability to model EBI maintenance and delivery over time [11]. This model suggests that EBIs should be sustained for a number of years, but only be sustained as long as an EBI continues to benefit to the population. It is difficult to determine *when* agencies, policymakers, and researchers might expect to see the benefits of an intervention, *when* EBIs might reach their maximum benefit, *who* the EBI benefits most, or *how often* to measure its impacts [5]. Our ABM demonstrated intervention benefits after at least 2 years, peaking after two or more additional years. However, if there were fewer human resource investments, evidence of benefits for both the overall and target populations was much slower. As such, assessments may need to be conducted at multiple years post implementation to assess continued benefit for the target population. We did not observe drastic differences in population benefit within a single year, suggesting the ideal interval for assessing RESPECT or a similar EBI's impact may be every 2 to 3 years. While these estimates are likely highly dependent on the specific EBI and context of delivery, our results suggest that in assessing sustainability, multiple year intervals may be most appropriate. This is in contrast to other phases of EBI implementation, like initial adoption, which often unfold in a series of months.

Furthermore, our ABM suggests that overall population benefit and target population benefits might become evident as well as differential. For example, individuals at greatest risk continued to benefit from the EBI over the entire 10 years over the simulation run, suggesting that rather than stopping an intervention entirely, it may be more appropriate to narrow eligibility criteria for an intervention to a more targeted portion of the population or adapt the intervention once the intervention has saturated a current population to be more resource-efficient. This supports the current strategy of many public health approaches, especially in HIV prevention, which targets those at highest risk, even among traditionally at-risk groups like men who have sex with men [43].

### The potential value of ABM for EBI sustainability

This study demonstrates how ABM can be a useful strategy in dissemination and implementation research to address the complex interactions among factors influencing EBI sustainability and intended population outcomes. Two commonly cited methodological challenges associated with examining EBI sustainability are (1) the amount of time needed to adequately follow EBIs and indications of population benefit and (2) the volume of data needed to adequately fit statistical hierarchical models as part of quantitative longitudinal designs [1]. Through ABM, we were able to circumvent these limitations, examine EBI sustainability over a much longer period of time (the equivalent of 10 years) than would have possible or feasible using conventional methods, and demonstrate how individual-level changes in a public health outcome gives rise to overall population-level characteristics. This is supported by our ABM results showing that while the overall mean population risk may seem to level out over time, the proportion of the population at high risk may continue to decrease. More investigations into how EBI sustainability influences different portions of the population, especially those most at risk, are needed to help justify or rethink initial resource allocation and timing and to limit the possibility that efforts do not exacerbate or create disparities in health outcomes.

To address these issues, ABMs build on theory to guide assumptions for future models as well as for informing longitudinal research designs. ABMs could be used to estimate which variables are likely most influential on the outcomes of interest and, as such, should be a priority for prospective data collection. For example, an emergent result of our ABM is evidence that there may not be additional benefit if the number of staff positions exceeds population need for an intervention. This may be counterintuitive, since the natural inclination is to believe that simply having more resources to address a public health problem is better. Although an excess of investment rarely seems to be the case in reality given that public health efforts are often underfunded, assessing optimal levels of support can ensure more effective use of resources. Additionally, ABMs could provide insight into key time points when effects of organizational-, program-, and individual-level variables will likely become evident in population outcomes. The value of ABMs for implementation research will be enhanced through the use of implementation frameworks to inform model development, identify key variables and relationships modeled, and justify model assumptions.

ABMs may also offer several practical benefits for practitioners and policy makers. There are increasing numbers of EBIs available for any particular health issue [1]. It is often difficult to assess how any EBI will fit with an agency, agency resources, community, or population [41]. ABMs simulating EBIs could help agencies assess, select, and adapt the most appropriate EBI to fit the local context. Similarly, ABMs may help policy makers compare how different EBIs or a combination of EBIs delivered at the local level influence population-level outcomes. For example, we modeled an HIV counseling intervention focused on reducing risk through behavior change. However, there are numerous approaches to HIV prevention including behavioral, biomedical, and structural EBIs that address different aspects of HIV risk. Many have debated the relative success of one type of HIV intervention approach over another (e.g., the success of behavioral interventions compared to the success of biomedical interventions) [44–46]. As others are beginning to demonstrate [23], ABMs, which model heterogeneous systems (e.g., different types of interventions), could be used to view how a collection of EBIs influences health outcomes.

### Limitations

ABM models represent features of real-world systems, but like models used in other scientific fields, they inherently reduce the complexity of such systems. Thus, the researcher must identify the essential components of the system and make simplifying assumptions about relationships that are related to the processes in question [47]. For example, we modeled a narrow set of organizational capacity variables and a relatively static client population that did not fluctuate in size over time. In reality, client populations change over time. Thus, our simulation does not incorporate the types of population dynamics that would naturally occur in a real context. We have focused on illustrating a specific set of relationships using a rather generic model of EBI implementation. While we extensively used theoretical and empirical literature to identify essential components, guide the simulation design, and make simplifying assumptions, we recommend conservative application of the results demonstrated in this model to specific circumstances and replication of our findings. Including more variables relevant to the sustainability of EBIs, such as funding to support intervention delivery or additional population dynamics, may impact the outcomes from our study and would be valuable extensions of the model.

## Conclusions

Adequate human resources, especially sufficient numbers of trained staff, are a key influence on EBI sustainability and population outcomes. However, the relationships among human resources, EBI sustainability, and population outcomes are dynamic and complex. Agent-based modeling helps to demonstrate these dynamic relationships and is an innovative tool for exploring aspects of organizational capacity, EBI sustainability, and population health in the future.

## Additional file

Additional file 1: A screen shot of the simulation view and code. (DOCX 992 kb)

## Abbreviations

ABM: Agent-based modeling
CDC: Centers for Disease Control and Prevention
EBI: Evidence-based interventions
HIV: Human Immunodeficiency Virus
