# Supplementary material for: The dynamic influence of human resources on evidence-based intervention sustainability and population outcomes: an agent-based modeling approach
Source: Implement Sci. 2018 Jun 5;13:77. doi: 10.1186/s13012-018-0767-0 (PMC5987464; doi:10.1186/s13012-018-0767-0)
Supplement: Supplementary file 1 — A screen shot of the simulation view and code. (DOCX 992 kb) [file 13012_2018_767_MOESM1_ESM.docx]

**Additional File 1**

**Screen Shot of User Interface**
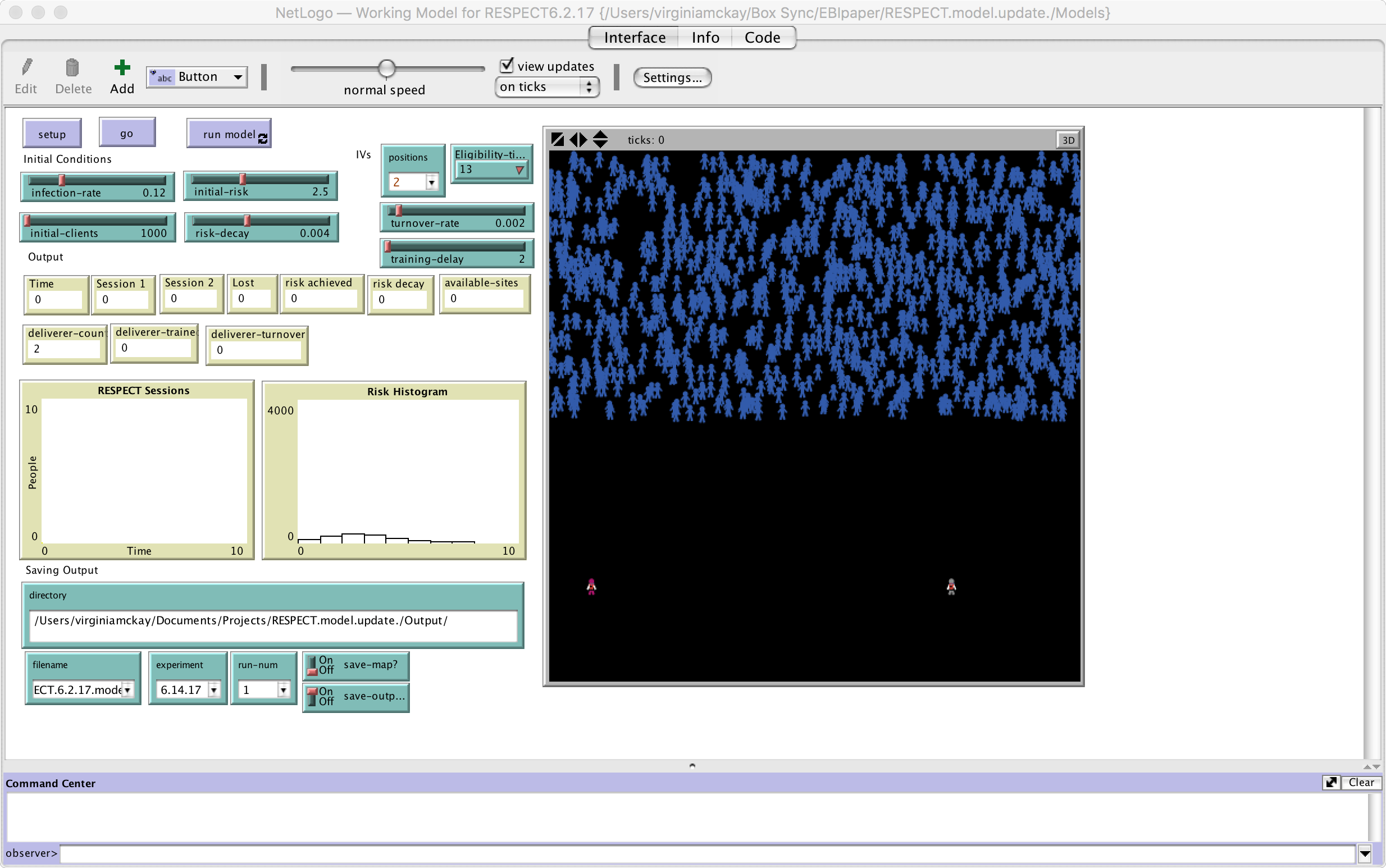


**Model Code**

breed [deliverers human] ;one set of deliverers

breed [clients human] ; one set of clients

globals [

work-days ; the number of "days", but will be represented by the number of times the model loops through the set of procedures. ticks is represented in weeks.

delivery-site-locations; an initial set of potential locations where RESPECT will be delivered. (10 total, locations fixed for all runs)

available-position ; the actual set of locations where RESPECT will be delivered based on the number positions specified in the input

;variables to relevant to clients

client-count ; the number of clients in the simulation

RESPECT2-count; the number of clients that have received both session of RESPECT

RESPECT1-count; the number of clients that have received the first session of RESPECT

total-lost; the number of clients that have been lost to follow up

;variables relevant to deliverers

deliverer-count;cumulative # of individuals hired

deliverer-trained; cumulative # of individuals that leave

deliverer-turnover; cumulative # of individuals that leave

;;;risk summary output;;

total-risk-achieved; the number of clients that acheive their risk reduction step

total-risk-decay; the number of clients that experience behavior decay

risk-mean ; average risk for the population

risk-max ; highest risk value in the populations

risk-min ; lowest risk value in the population

risk-median ; median risk value in the population

risk-0 ; number of indiviuals with a risk value <.05

risk-1 ; number of individuals with a risk value >=.05 and >1.5

risk-2 ; number of individuals with a risk value >=1.5 and >2.5

risk-3 ; number of individuals with a risk value >=2.5 and >3.5

risk-4 ; number of individuals with a risk value >=3.5 and >4.5

risk-5 ; number of individuals with a risk value >=4.5 and >5.5

risk-6 ; number of individuals with a risk value >=5.5 and >6.5

risk-7 ; number of individuals with a risk value >=6.5

]

; variables specific to each individual client in the population

clients-own [ ; eventually these will be variables for each of the clients. These are outcome variables that were tracked as part of the original RESPECT RCT.

;infected? ; HIV infection

risk; the level of risk that the individuals have

session-one? ; receiving the initial session

session-two? ; if the person will return for the second session of the program

lost?; if the person is lost to follow up

risk-achieved?

risk-decay? ; if the person has experienced risk decay after receiving RESPECT

age

]

;variables specific to each individual deliverer in the model

deliverers-own [

age

trained?]

patches-own [

available?

empty-time]; variable that will count the number of delivery sites that do not have capacity

;-----setup procedures-------;

to setup

clear-all

setup-globals ; sets up all global variables

setup-deliverysites ; sets up the locations for RESPECT delivery

setup-people ; procedure that creates the client population and "shapes them"

set client-count (count clients) ; creates a variable that keeps track of the number of potential clients

set RESPECT2-count (count clients with [session-two? = true]) ; creates a variable that keeps track of the number of clients that have received both session of RESPECT.

set RESPECT1-count (count clients with [session-one? = true]) ; creates a varible that keeps track of the number of clients that have received the first sesion of RESPECT.

set total-lost (count clients with [lost? = true])

setup-clientsown; procedure that randomly assigns infection and risk among the number of clients

setup-deliverersown; procedure that sets up variables for deliverers

set total-risk-achieved 0

set total-risk-decay 0

set deliverer-count (count deliverers)

set deliverer-trained 0

set deliverer-turnover 0

reset-ticks; sets the time at zero

if save-output? = true [update-output print-output]

end

to go

if not any? clients with [session-two? = false] or ticks = 500 [stop]; stops the model from running if every client has received both sessions of RESPECT

;if ticks = 50 [stop]

ifelse work-days = 15 [ ; procedure for moving clients to the RESPECT sites and loops through the procedures 6 times. I did this because the counselors report -->

set work-days 0 ; that they see on average 6 clients a week.

update-deliverer-capacity

update-elligibility

update-behavior-decay

tick

if save-output? = true ;and remainder ticks 50 = 0

[

update-output

print-output]]; represents a week

[recruit

deliver-RESPECT ; procedure for receiving the intervention and tracking deliverer delivery

returntopop ; procedure for moving clients back into the population

set work-days (work-days + 1)

]; counts the number of work days that have past.

end

;;;;;;;;;;;;;;;;;;;;;;;;;;;;;;;;;;;;;;;;;;;;;;;;;

;;all of the set up procedures are listed below;;

;;;;;;;;;;;;;;;;;;;;;;;;;;;;;;;;;;;;;;;;;;;;;;;;;

to setup-globals

set delivery-site-locations (patch-set

patch -13 -10 patch -10 -10 patch -7 -10 patch -4 -10 patch -1 -10 patch 2 -10 patch 5 -10 patch 8 -10 patch 11 -10 patch 14 -10

patch -13 -13 patch -10 -13 patch -7 -13 patch -4 -13 patch -1 -13 patch 2 -13 patch 5 -13 patch 8 -13 patch 11 -13 patch 14 -13); locations where the deliverers and clients will move for RESPECT delivery

;ask delivery-site-locations [set pcolor blue]

set work-days 0

end

to setup-deliverysites ; to set up the delivery site locations.

ask patches ; Set up the paches own variables

[set empty-time 0

set available? false]

ask n-of positions delivery-site-locations ; ask patches in the patch set defined as delivery-site locations to set available true based on the number of positions specifcied in the input

[set available? true] ;

set available-position (patch-set patches with [available?] ) ; create a new patch set based on the patches with this true variable.

end

to setup-people

create-clients initial-clients ; set up an initial number of clients based on initial-clients slider in the interface

ask clients [setxy random-xcor random-float max-pycor ; set all of the population characteristics

set shape "person"

set heading 180

set color blue

]

create-deliverers positions ; set up an initial number of clients based on an initial deliverer-num slider in the interface

ask deliverers [ ; set all of the deliverer characteristics

set shape "person service"

set heading 0

move-to one-of available-position while [any? other turtles-here] [move-to one-of available-position]] ; move to one of the available positions defined above

end

to setup-clientsown ; set up client characteristics

ask clients[

;set infected? (who < client-count * infection-rate) ; number of clients are assinged an infection based on the initial infection rate; infection rate is on a slider

set risk random-poisson initial-risk ; sets a risk level for an individual by randomly selecting a number from a poisson distribution with the mean as a slider in the interface (is infected? unrelated to risk? Shouldn't infection be related to risk?)

if risk > 7 [set risk 7] ; if by chance the indivdiual has a risk higher than seven, the it is reassigned a 7 to create a cap.

if risk < 0 [set risk 0]

;ifelse infected? [set color yellow] [set color blue] ; if the person is HIV+, set their color as yellow

set session-one? false ; all clients start without having the 1st session of RESPECT

set session-two? false ; all clients start without having the 2nd session of RESPECT

set lost? false

set risk-achieved? false ; all clients start without having achieved their risk reduction step

set risk-decay? false ; no clients start with having experience risk decay

set age 0

]

end

to setup-deliverersown ; set up deliverer characteristics

ask deliverers[ ; all deliverers start at an age of 0 and without training

set age 0

set trained? false

]

end

;;;;;;;;;;;;;;;;;;;;;;;;;;;;;;;;;;;;;;

;;ALL go procedures are listed below;;

;;;;;;;;;;;;;;;;;;;;;;;;;;;;;;;;;;;;;;

;to recruit procedure starts by asking clients that have received session 1 to return for their follow up appointment. Then fill the remaining spaces new clients

to recruit

ask clients with [session-one? = true and session-two? = false and lost? = false] ;recruit session 1 peeps that haven't had the second session yet.

[if random-number < .90 and available-sites > 0 ;number of clients sites and emptysites based on the number of followup clients that counselors report per week; set so that there is about 89% loss to follow up after a year.

[move-to one-of available-position while [any? other clients-here] [move-to one-of available-position]] ; move to a delivery site

]

ask clients with [session-one? = false] ; recruit new clients

[if random-number < .001 and available-sites > 0 ; pick however many clients and then move them.

[move-to one-of available-position while [any? other clients-here] [move-to one-of available-position]]] ; move to a delivery site

end

to-report random-number ; random number reporter used to select followup and new clients from the population; also used to decrease risk among clients below

report (random-float 1)

end

to-report available-sites ; reported to count the number of client delivery sites that are empty and "available"; used to determine capacity to deliver RESPECT to clients

report (count available-position with [not any? clients-here and any? deliverers-here with [trained?]])

end

to-report trained-deliverers

report (count deliverers with [trained? = true])

end

; to deliver-RESPECT procedure simulates a client "receiving the RESPECT intervention. It starts by changing the RESPECT variables from false to true

; i.e., if session one is false, change to true, if session one is true, the change session one to false)

to deliver-RESPECT

ask clients-on available-position

[ifelse session-one? ;

[set session-two? true

set color green] ; if session one is true, set session two true

[

set session-one? true ; otherwise, set session one true, and assign .85 lost to follow up

set color yellow

if random-number > .85

[set lost? true]

]]

ask clients-on available-position; increases the likelihood that someone will reduce their risk.

[ifelse session-two? and random-number > .28 [

set risk (risk - (random-normal 1 .5)) ;

set risk-achieved? true

set total-risk-achieved (total-risk-achieved + 1)] []

if risk > 7 [set risk 7] ; if by chance the indivdiual has a risk higher than seven, the it is reassigned a 7 to create a cap.

if risk < 0 [set risk 0]] ; This is supposed to simulate the risk reduction step --> 70% of individuals will successfully reduce their risk. (Risk will be reduced by subtracting .5. Could this lead to negative risk, or is there a check elsewhere to prevent values from falling below 0? Multiplying risk by some fraction would be a way to avoid negative values.)

end

to returntopop ;ask clients to move back into the general population

ask clients-on available-position [

setxy random-xcor random-float max-pycor]

end

;procedure that simulates deliverer "capacity" by incoporating training for deliverers if they've been at a site for

; a certain amount of time and turnover among deliverers that have been there for at least two years.

to update-deliverer-capacity ; procedure for updating the capacity to deliver RESPECT at the end of the work week

ask deliverers ; updates variables for deliverers, increases age for every week worked

[ set age (age + 1)

if age = training-delay

[set trained? true

set deliverer-trained (deliverer-trained + 1) ] ; after four weeks the deliverer becomes trained.

if random-number < turnover-rate [ ; over a period of time the counselor will turnover

set deliverer-turnover (deliverer-turnover + 1)

die

] ;set deliverer-turnover (

]

ask available-position ; creates new deliverers after empty spaces have been vacant for a period of time.

[if not any? deliverers-here [set empty-time (empty-time + 1)]

if empty-time > 4 [sprout-deliverers 1 [

set age 0

set trained? false

set shape "person service"

set heading 0]

set deliverer-count (deliverer-count + 1)]

if empty-time > 4 [set empty-time 0]]

end

to update-elligibility ; this procedure resets elligibility for participation in RESPECT and tracks how long it's been since participating in the first session of RESPECT.

ask clients with [(session-one? = true and age = Eligibility-time and lost? = true and risk > 4) or (session-two? = true and age = Eligibility-time and risk > 4)]

[

set session-one? false

set session-two? false

set risk-decay? false

set risk-achieved? false

set lost? false

set age 0

set color blue

]

ask clients with [(session-two? = true and age < 25) or (session-one? = true and age < 25 and lost? = true)]

[set age (age + 1)]

end

to update-behavior-decay ; this procedure randomly selects indivdiuals to loose their risk reduction behavior according RESPECT RCT.

ask clients with [session-two? = true and risk-achieved? = true and risk-decay? = false]

[if random-number < risk-decay

[set risk (risk + (random-normal 1 .5))

set risk-decay? true

set total-risk-decay (total-risk-decay + 1)] ; The risk-decay value is high enough in this default that the net result is that people who receive RESPECT are more likely than those who don't to increase their risk.

if risk > 7 [set risk 7] ; if by chance the indivdiual has a risk higher than seven, the it is reassigned a 7 to create a cap.

if risk < 0 [set risk 0]] ; if by chance the individual has a risk lower than one, then it is reassigned a 0 to created a floor.

end

to update-output

set RESPECT2-count (count clients with [session-two? = true]) ; creates a variable that keeps track of the number of clients that have received both session of RESPECT.

set RESPECT1-count (count clients with [session-one? = true]) ; creates a varible that keeps track of the number of clients that have received the first sesion of RESPECT.

set total-lost (count clients with [lost? = true]); creates a variable that keeps track of the number of clients that have been lost to follow up

set risk-mean mean [risk] of clients ; average risk for the population

set risk-max max [risk] of clients; highest risk value in the populations

set risk-min min [risk] of clients ; lowest risk value in the population

set risk-median median [risk] of clients ; median risk value in the population

set risk-0 count clients with [risk < .5]; number of indiviuals with a risk value <= 1

set risk-1 count clients with [risk >= .5 and risk < 1.5]; number of individuals with a risk value >1 and =2

set risk-2 count clients with [risk >= 1.5 and risk < 2.5]; number of individuals with a risk value >2 and =3

set risk-3 count clients with [risk >= 2.5 and risk < 3.5]; number of individuals with a risk value >3 and =4

set risk-4 count clients with [risk >= 3.5 and risk < 4.5]; number of individuals with a risk value >4 and =5

set risk-5 count clients with [risk >= 4.5 and risk < 5.5]; number of individuals with a risk value >5 and =6

set risk-6 count clients with [risk >= 5.5 and risk < 6.5]; number of individuals with a risk value >6

set risk-7 count clients with [risk >= 6.5]; number of individuals with a risk value = 7

end

;;;;;;;;;;;;;;;;;;;;;;;;;;;;;;;;;

;;------output procedures------;;

;;;;;;;;;;;;;;;;;;;;;;;;;;;;;;;;;

to print-map

set-current-directory directory

if save-map? [export-view (word "map" filename "(" experiment ")(" run-num ").png")]

end

to setup-output ; setting up a .csv file

file-open (word directory filename "(" experiment ").csv") ;

file-type (word "independent variables, time, positions, turnover-rate, training-delay, elligibility-time,") ; independent variables

file-type (word "monitors, deliverer-count, deliverer-trained, deliverer-turnover, available-sites,"); variables for monitoring, calibration, and validation purposes

file-type (word "dependent variables, session1, session2, lost-to-followup, total-risk-achieved, total-risk-decay, risk-mean, risk-max, risk-min, risk-median, risk-0, risk-1, risk-2, risk-3, risk-4, risk-5, risk-6, risk-7 ") ;; dependent variables

file-print ""

file-close

end

to print-output ; setting up a .csv file

file-open (word directory filename "(" experiment ").csv")

file-type (word "," ticks "," positions "," turnover-rate "," training-delay "," Eligibility-time ","); independent variables

file-type (word "," deliverer-count "," deliverer-trained "," deliverer-turnover "," available-sites ",") ; variables for monitoring, calibration, and validation purposes

file-type (word "," RESPECT1-count "," RESPECT2-count "," total-lost "," total-risk-achieved "," total-risk-decay "," risk-mean "," risk-max "," risk-min "," risk-median "," risk-0 "," risk-1 "," risk-2 "," risk-3 "," risk-4 "," risk-5 "," risk-6 ", " risk-7 ",") ;; dependent variables

file-print "" ; creates a hard return in the csv file

file-close

end
